# Effects of Rikkunshito on postgastrectomy weight loss and nutritional status in gastric cancer patients: A retrospective observational study

**DOI:** 10.1097/MD.0000000000043950

**Published:** 2025-08-15

**Authors:** Kenichi Iwasaki, Edward Barroga, Yuki Takano, Toru Sakurai, Erika Yamada, Masaya Enomoto, Yota Shimoda, Junichi Mazaki, Hiroyuki Koga, Akishige Kanazawa, Yuichi Nagakawa

**Affiliations:** a Department of Gastrointestinal and Pediatric Surgery, Tokyo Medical University, Tokyo, Japan; b Medical English Education Center, Showa Medical University, Tokyo, Japan.

**Keywords:** gastric cancer, nutritional status, postgastrectomy, rikkunshito, weight loss

## Abstract

Postgastrectomy disorders decrease the quality of life of patients because of poor oral intake and nutritional status. Rikkunshito is a Japanese herbal medicine that alleviates anorexia and prevents upper gastrointestinal disorders, particularly in the perioperative period. Herein, we investigated whether Rikkunshito administration in the early perioperative period alleviates weight loss and improves the nutritional status after undergoing minimally invasive distal gastrectomy for gastric cancer. We conducted a retrospective cohort study involving 139 consecutive gastric cancer patients who underwent laparoscopic or robot-assisted distal gastrectomy for potentially curable gastric cancer between January 2018 and May 2023 at our institution. We divided the patients into 2 groups based on Rikkunshito administration time: Rikkunshito administered on postoperative day 4 (RPOD4 group) and on postoperative day 1 (RPOD1 group). We performed one-to-one propensity score matching to balance the baseline characteristics. We examined the postoperative body weight changes and nutritional status at 1 month. One month postoperatively, the RPOD1 group showed a significantly smaller body weight change rate (7.12% [RPOD4] vs 5.35% [RPOD1], *P* < .05). For nutritional status, the RPOD1 group showed significantly higher prognostic nutritional index score (47.21 ± 16.45 [RPOD4] vs 49.45 ± 5.50 [RPOD1], *P* < .05) and geriatric nutritional risk index score (97.22 ± 20.49 [RPOD4] vs 100.97 ± 7.96 [RPOD1], *P* < .05). Initiating rikkunshito in the early perioperative period alleviates weight loss and improves the nutritional status after minimal invasive distal gastrectomy for gastric cancer.

## 1. Introduction

Gastric cancer is the 5th most common type of cancer and remains a significant global health burden, necessitating effective therapeutic strategies to improve patient outcomes.^[1]^ Surgical resection is the cornerstone in the management of gastric cancer. However, complications usually occur in the postoperative period, including a compromised nutritional status and decreased quality of life. These complications underscore the importance of a comprehensive perioperative care to address not only the primary disease but also the secondary effects of surgery on the well-being of patients.

Postoperative complications on the nutritional status of gastric cancer patients have been well-reported.^[2–4]^ Weight loss and malnutrition following gastric resection are associated with adverse outcomes, including impaired wound healing, increased infection risk, and compromised long-term survival.^[5,6]^ As such, interventions aimed at preserving the nutritional status and supporting recovery during the perioperative period are of paramount importance.

Traditional Japanese herbal medicines, such as Rikkunshito (Tsumura, Tokyo, Japan), have attracted attention for their potential to mitigate postoperative complications and improve patient outcomes.^[7]^ Rikkunshito has shown efficacy for postgastrectomy complications, particularly in enhancing gastrointestinal motility and preventing stasis.^[8,9]^ In addition to its prokinetic and antireflux properties, Rikkunshito has been reported to enhance ghrelin secretion, stimulate appetite, and improve food intake, particularly in patients with upper gastrointestinal disorders or cancer-related anorexia.^[10–12]^ These physiological effects suggest that Rikkunshito could potentially attenuate postoperative weight loss and malnutrition through endocrine and functional pathways.

Despite these promising mechanisms, the broader effects of Rikkunshito on weight loss and overall nutritional status of patients undergoing distal gastrectomy for gastric cancer remain an area of active investigation. Moreover, the clinical utility of Rikkunshito in the early perioperative period following minimally invasive distal gastrectomy has not been well established.^[13,14]^ Therefore, in this study, we investigated whether the early perioperative administration of Rikkunshito could mitigate postoperative weight loss and improve the nutritional status of patients after undergoing minimally invasive distal gastrectomy for gastric cancer.

## 2. Methods

### 2.1. Participants

**Figure 1.**
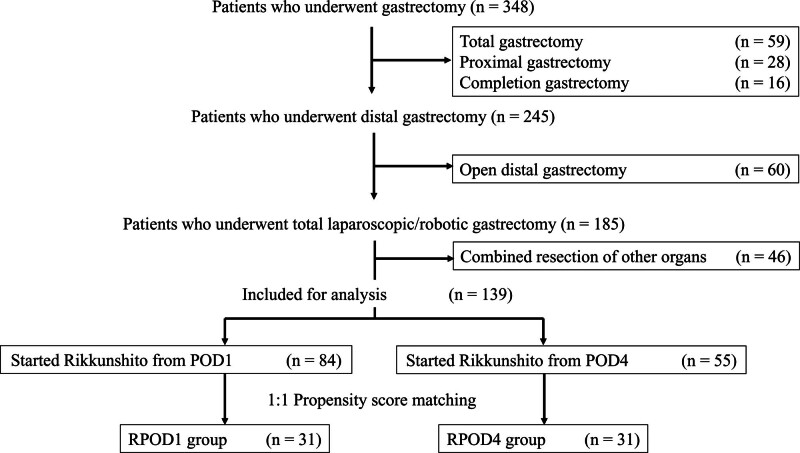
Flow diagram illustrating the selection process of the 139 patients included in the final analysis, detailing initial enrollment, exclusion criteria, and the application of PSM. PSM = propensity score matching.

A total of 139 consecutive patients who were pathologically diagnosed with gastric cancer were assessed in this retrospective cohort study. The patients underwent minimal invasive gastrectomy (i.e., laparoscopic or robot-assisted distal gastrectomy) for potentially curable gastric cancer between January 2018 and May 2023 at our institution. Curative gastrectomy with lymphadenectomy was routinely performed in all the patients. The inclusion criterion was patients with resectable gastric cancer. The exclusion criteria were as follows: patients without recorded postoperative body weight (BW); patients with concurrent malignancy; patients with recurrent or metastatic cancer; patients with major organ dysfunction (e.g., cardiac failure or acute inflammatory disease); patients unable to take oral medications; and patients with preexisting nutritional disorders or contraindications to Rikkunshito. The flow diagram of the inclusion of the 139 patients is shown in Figure 1.

### 2.2. Study design

The 139 patients were divided into 2 groups based on the timing of Rikkunshito administration. The RPOD4 group (n = 55) received Rikkunshito from the 4th postoperative day (POD4). The RPOD1 group (n = 84) were started on Rikkunshito from the first postoperative day (POD1). Rikkunshito was administered orally according to established dosage guidelines. The patients received 2.5 g of Rikkunshito before each meal (a total of 7.5 g per day) for 1 month after undergoing distal gastrectomy. The study was conducted in accordance with the dosage and administration approved for Rikkunshito in Japan (the usual adult dose is 7.5 g/day orally in 2 or 3 divided doses before or between meals). The only difference between the 2 groups was the timing of initiation: the early group started on POD 1, whereas the delayed group started on POD 4. Consequently, patients in the early group received Rikkunshito for 3 more days (22.5g) in total. This design was intended to assess whether earlier administration of Rikkunshito could contribute to better prevention of postoperative weight loss and maintenance of nutritional status. The initiation of postoperative enteral feeding of all patients included in the analysis for both groups was on POD4.

Rikkunshito (Tsumura, Tokyo, Japan) is a Kampo extract formulation prepared by decocting 8 crude drugs (Hoelen [18.6%], Ginseng radix [18.6%], Atractylodis lanceae rhizoma [18.6%], Pinelliae tuber [18.6%], Zizyphi fructus [9.3%], Aurantii nobilis pericarpium [9.3%], Glycyrrhizae radix [4.7%], and Zingiberis rhizoma [2.3%]) with water alone, followed by spray-drying to produce a dry extract, which is then processed into granules using a dry granulation method without the use of any organic solvents or additional water. More detailed descriptions of all these components were reported previously.^[15]^

### 2.3. Surgical procedures

Surgery was performed by R0 minimal invasive distal gastrectomy with sufficient lymphadenectomy. The duodenal bulb was transected, and 2-thirds of the stomach was dissected, following the Japanese Gastric Cancer Treatment Guidelines.^[16]^

### 2.4. Measurement of outcomes

The short-term outcomes were perioperative changes in body weight analyzed by percent change and absolute change, operative time, blood loss, length of hospital stay, and postoperative complications.

The perioperative nutritional status was assessed using prognostic nutritional index (PNI), geriatric nutritional risk index (GNRI), glasgow prognostic score (GPS), modified GPS (mGPS), neutrophil/lymphocyte ratio, platelet/lymphocyte ratio, and CRP/albumin ratio (CAR), and were compared between the 2 groups. These parameters were measured before surgery and 1 month postoperatively. The assessment of postoperative nutritional intake was performed quantitatively, with total intake assigned a score of 100 and no intake assigned a score of 0. All oncological definitions were in accordance with the Japanese Classification of Gastric Carcinoma 15th Edition.^[17]^

### 2.5. Statistical analysis

Propensity score matching (PSM) was performed to balance the baseline characteristics and overcome possible patient selection bias between the RPOD4 and RPOD1 groups owing to the retrospective study design. A regression model was created based on potential variables. The selected covariables included age, gender, body mass index, American Society of Anesthesiologists physical status, history of neoadjuvant chemotherapy, clinical tumor, node, and metastasis stage, operation type, extent of lymphadenectomy, and combined resection.

To match the propensity scores, a 1:1 nearest neighbor matching algorithm with an optimal caliper width of 0.2 without replacement was applied. Descriptive statistics were presented as means ± standard deviations for continuous variables and as frequencies for categorical variables. The Student *t*-test and chi-square test were used for continuous and categorical variables, respectively. Statistical analysis was performed using SPSS 13.0 software and G*Power software version 3.1.9 (Heinrich-Heine-Universität Düsseldorf, Düsseldorf, Germany). A *P*-value < .05 was considered to indicate a statistically significant difference.

### 2.6. Ethics and informed consent

The Ethics Committee of Tokyo Medical University approved the study protocol (Approval No. T2019-0462) and waived the requirement for informed consent for the use of anonymized patient data. This study conforms to the provisions of the Declaration of Helsinki. All information was collected after obtaining written informed consent from the participants.

## 3. Results

### 3.1. Patient selection and baseline characteristics

A total of 139 patients who underwent gastrectomy were enrolled in the study. Among them, 55 patients received Rikkunshito starting on postoperative day (POD) 4 (RPOD4 group), and 84 patients received Rikkunshito from POD1 (RPOD1 group). After PSM, 31 patients were included in each group for further analysis. The baseline clinicopathological characteristics of the matched cohorts are summarized in Table 1. No significant differences were observed between the 2 groups in terms of age, sex, body mass index, tumor stage, surgical approach, or comorbidities.

**Table 1:** Clinicopathological characteristics of propensity score-adjusted patients.

Characteristics		RPOD4 (n = 31)	RPOD1 (n = 31)	*P*-value
Age (year)	Median; range	71 (40–82)	67 (47–88)	.687
Sex	Male/Female	23/8	25/6	.551
Body mass index (kg/m^2^)				
	Median; range	22.7 (17.5–33.9)	23.5 (18.3–30.1)	.716
ASA-PS	(1/1<)	15/16	16/15	.803
cT category	(1/2/3/4)	26/4/1/0	25/6/0/0	.902
cN category	(0/1/2/3)	30/1/0/0	30/1/0/0	1
Clinical TNM stage	(I/IIA/IIB/III)	30/0/1/0	30/1/0/0	.644
(JCGC 15th edition)				
Tumor size (mm)	Median; range	23 (12–80)	30 (4–70)	.549
Tumor location	(upper/middle/lower)	0/22/9	17/14	.194
Histologic type	(Differentiated/Undifferentiated)	12/19	15/16	.908
Neoadjuvant chemotherapy	(±)	0/31	0/31	1
Extent of lymphadenectomy				
–	(Less than D2/D2 or more)	26/5	23/8	.358

ASA-PS = American Society of Anesthesiologists physical status, JCGC = Japanese Classification of Gastric Carcinoma, RPOD1 = Rikkunshito administered on postoperative day 1, RPOD4 = Rikkunshito administered on postoperative day 4, TG = total gastrectomy, TNM = tumor, node, and metastasis.

### 3.2. Preoperative nutritional status

Preoperative laboratory evaluations revealed no significant differences between the RPOD4 and RPOD1 groups in terms of nutritional markers. Specifically, serum albumin levels, total lymphocyte counts, and other indicators of nutritional status were comparable. Detailed data on preoperative nutritional parameters are shown in Table 2.

**Table 2:** Nutritional parameters of propensity score-adjusted patients.

Characteristics		RPOD4 (n = 31)	RPOD1 (n = 31)	*P*-value
NLR	(Mean; ±SD)	2.039 ± 0.993	3.070 ± 4.500	.237
PLR	(Mean; ±SD)	204.5 ± 43.72	235.1 ± 288.1	.700
CAR	(Mean; ±SD)	0.062 ± 0.213	0.057 ± 0.087	.927
PNI	(Mean; ±SD)	47.28 ± 4.143	49.05 ± 5.504	.635
GNRI	(Mean; ±SD)	99.85 ± 20.47	103.9 ± 8.592	.059
GPS	(Mean; ±SD)	0.065 ± 0.250	0.069 ± 0.258	.946
mGPS	(Mean; ±SD)	0.065 ± 0.250	0.129 ± 0.341	.399
CONUT	(Mean; ±SD)	1.258 ± 1.032	1.419 ± 1.177	.410

CAR = CRP/albumin ratio, CONUT = controlling nutritional status, GNRI = geriatric nutritional risk index, GPS = glasgow prognostic score, mGPS = modified GPS, NLR = neutrophil/lymphocyte ratio, PLR = platelet/lymphocyte ratio, PNI = prognostic nutritional index, RPOD1 = Rikkunshito administered on postoperative day 1, RPOD4 = Rikkunshito administered on postoperative day 4, SD = standard deviation.

### 3.3. Short-term surgical and postoperative outcomes

Surgical procedures and immediate postoperative outcomes did not differ significantly between the 2 groups. The length of surgery, intraoperative blood loss, and postoperative hospital stay were similar. The incidence of postoperative complications, including anastomotic leakage, delayed gastric emptying, and infectious complications, was also comparable (Table 3). There were no patients who required reoperation, and there was no in-hospital mortality in either group. Rikkunshito was well-tolerated in all patients, with no recorded adverse events related to the medication during the 1-month postoperative period.

**Table 3:** Short-term surgical and postoperative outcomes of overall and propensity score-adjusted patients.

Outcomes	RPOD4 (n = 31)	RPOD1 (n = 31)	*P*-value
Operative time (min) Median; range	361 (239–473)	343 (233–491)	.615
Estimated blood loss (mL) Median; range	28 (0–491)	43 (0–230)	.823
Number of dissected lymph nodes Median; range	39 (18–79)	34 (19–63)	.167
Postoperative hospital stay (d) Median; range	10 (9–21)	10 (6–19)	.797
Postoperative complications* n (%)	1 (3.23)	1 (3.23)	1
Reoperation/mortality during hospitalization	0	0	−

RPOD1 = Rikkunshito administered on postoperative day 1, RPOD4 = Rikkunshito administered on postoperative day 4.

* Clavien–Dindo classification grade ≥ 2, within 30 d after surgery.

### 3.4. Postoperative body weight change

**Figure 2.**
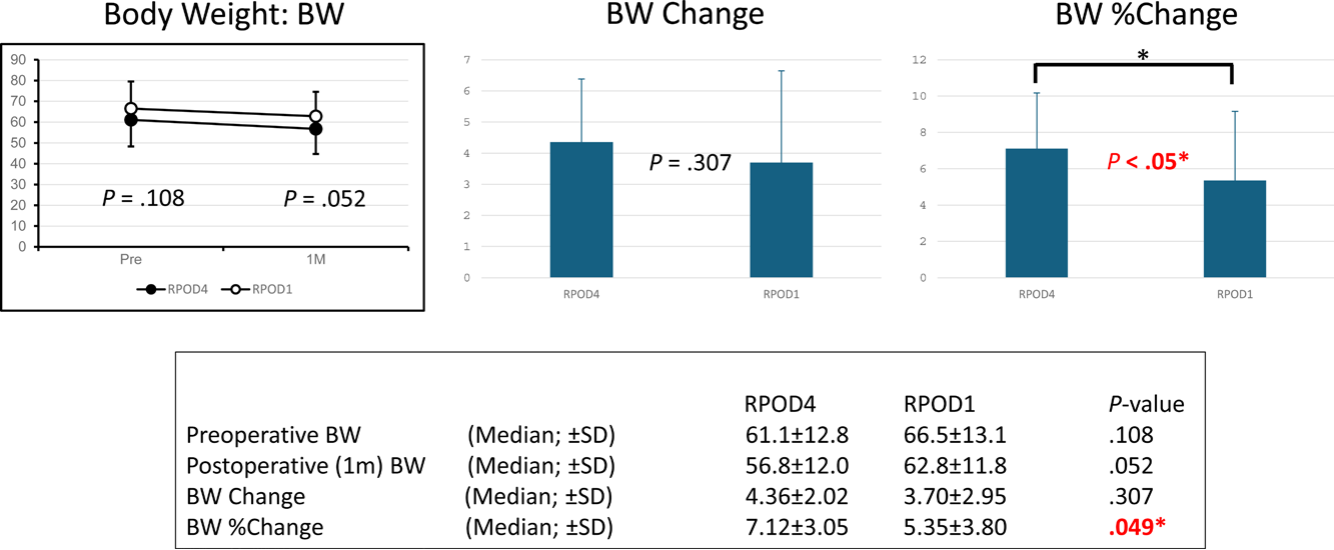
Comparison of postoperative BW, absolute BW change, and percentage change in BW from baseline to 1 month after surgery in the Rikkunshito and control groups. BW = body weight.

One month after surgery, although there was no significant difference in absolute body weight [56.8 kg (RPOD4) vs 62.8 kg (RPOD1), *P* = .05] or total body weight change [4.36 kg (RPOD4) vs 3.70 kg (RPOD1), *P* = .307], the percentage of body weight loss was significantly lower in the RPOD1 group [7.12% (RPOD4) vs 5.35% (RPOD1), *P* < .05], indicating a better preservation of body mass (Fig. 2).

### 3.5. Postoperative nutritional assessment

**Figure 3.**
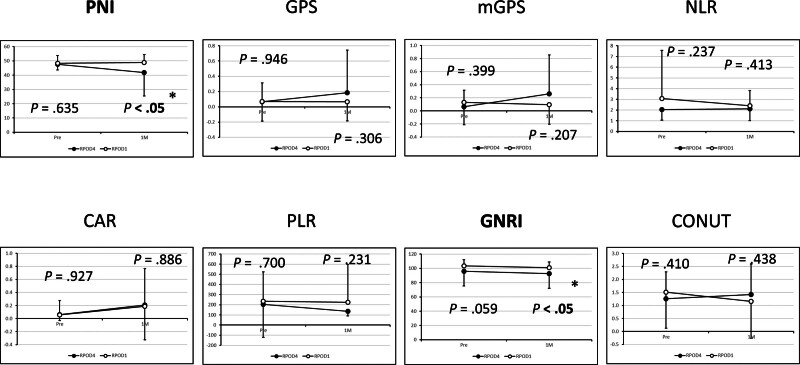
Assessment of nutritional status 1 month postoperatively, as evaluated using serum albumin level, total lymphocyte count, and the calculated PNI, with comparisons between the 2 groups. PNI = prognostic nutritional index.

Evaluation of postoperative nutritional status using multiple scoring systems revealed no significant differences in GPS, modified GPS (mGPS), neutrophil/lymphocyte ratio, C-reactive protein/albumin ratio (CAR), platelet/lymphocyte ratio, and controlling nutritional status (CONUT) score between the 2 groups. However, the PNI score was significantly higher in the RPOD1 group [49.45 ± 5.50] than in the RPOD4 group [47.21 ± 16.45], *P* < .05. Similarly, the GNRI score was significantly improved in the RPOD1 group [100.97 ± 7.96] vs RPOD4 group [97.22 ± 20.49], *P* < .05 (Fig. 3).

### 3.6. Postoperative food intake

**Figure 4.**
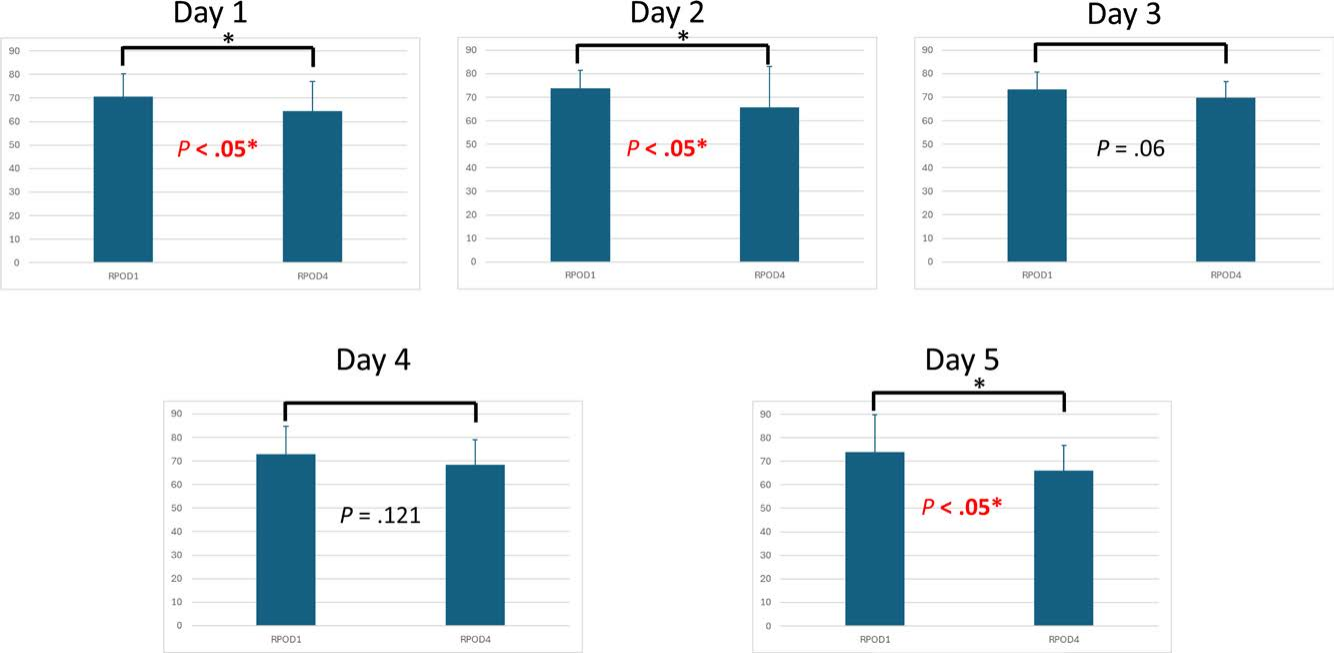
Postoperative oral intake over the first 5 days following the initiation of enteral feeding, comparing mean daily caloric intake between the Rikkunshito and control groups.

Quantitative assessment of oral intake after the initiation of enteral feeding revealed a significantly higher food intake in the RPOD1 group. On postoperative day 1 [64.52 ± 12.61 (RPOD4) vs 70.64 ± 9.63 (RPOD1), *P* < .05], day 2 [65.81 ± 17.28 vs 73.87 ± 7.61, *P* < .05], and day 5 [66.13 ± 10.54 vs 73.87 ± 15.85, *P* < .05], patients in the RPOD1 group consistently showed greater food consumption. The trend of oral intake over the 5-day period following the initiation of feeding is illustrated in Figure 4.

## 4. Discussion

In this study, we set out to investigate whether the early administration of Rikkunshito can prevent weight loss and maintain nutritional status by increasing oral intake in the critical postoperative period. Our results showed that the initiation of Rikkunshito in the early perioperative period alleviates the weight loss and improves the nutritional status of gastric cancer patients after minimal invasive distal gastrectomy.

Up to 55% of patients usually suffer from major appetite loss after abdominal surgery, even with enhanced recovery programs.^[18]^ Appetite loss that may lead to severe weight loss is a well-documented common phenomenon, particularly after upper gastrointestinal surgery.^[19]^ Thus far, weight loss and malnutrition after gastrectomy remain as inevitable and serious complications. Postoperative digestive symptoms including appetite loss is directly linked to the quality of life of patients who have undergone gastrectomy for gastric cancer, mainly resulting from maldigestion and delayed gastric emptying.^[20,21]^ Moreover, malnutrition from postoperative appetite loss may adversely impact survival and postoperative complications.^[22]^ The use of the ERAS protocol for early recovery after surgery, a concept originally used for colorectal surgery,^[23]^ has also been applied to gastrectomy to address this challenging problem. However, the ERAS protocol was not efficacious. In the present study, early postoperative administration of Rikkunshito was associated with improved oral intake and a lower rate of weight loss. These findings suggest that Rikkunshito may play a supportive role in preserving postoperative nutritional status in patients undergoing minimally invasive gastrectomy. Importantly, this benefit may contribute directly to maintaining the quality of life of patients, which is frequently compromised by postoperative appetite loss and malnutrition. Our results are in line with previous studies reporting that Rikkunshito enhances gastrointestinal motility and appetite through ghrelin-mediated pathways.^[24,25]^ However, our study is distinct in that it demonstrates these effects when Rikkunshito is initiated in the early postoperative period, a strategy that has been insufficiently investigated in the context of minimally invasive surgery for gastric cancer. This early intervention approach may offer a meaningful enhancement to current perioperative care protocols, particularly where ERAS has shown limited benefit.

Rikkunshito is a traditional Japanese herbal medicine that is also known as Kampo medicine. It has been used for decades for treating various gastrointestinal conditions. Rikkunshito is derived from a mixture of 8 medicinal herbs and is known to stimulate appetite, aid digestion, and alleviate symptoms such as nausea and vomiting. Research on Rikkunshito has started to attract attention worldwide because of its promising results in improving symptoms associated with gastrointestinal disorders.^[26,27]^ In their systematic review, Wagner et al explicitly stated the relevance of Rikkunshito as a treatment option for those with appetite loss after a major abdominal surgery.^[28]^ Previous studies have reported the positive effects of Rikkunshito for reducing perioperative gastrointestinal symptoms postgastrectomy.^[26,29]^ The present study focused on assessing whether the timing of Rikkunshito intake has an effect on the body weight and nutritional status of gastric cancer patients postgastrectomy.

Davis et al reported that weight loss was particularly pronounced 1 month postgastrectomy, with subsequent stabilization thereafter.^[30]^ Therefore, we assessed whether there would be a significant difference in body weight and nutritional status 1 month postgastrectomy when Rikkunshito was administrated at different time periods, namely, POD1 and POD4. Additionally, in the present study, the duration of Rikkunshito administration was limited to the perioperative period, specifically within 1 month after surgery. Notably, the present results showed not only a significantly smaller rate of body weight loss but also a significant difference in PNI and GNRI. Both results are believed to be due to the increase in postoperative food intake, which was possibly influenced by Rikkunshito, enhancing intestinal peristalsis and motility in the RPOD1 group. Previous studies have reported the clinical benefits of Rikkunshito administration postgastrectomy, including minimization of body weight loss and improvement of the nutritional status.^[26,31]^ To the best of our knowledge, the present study is the first to report not only on Rikkunshito administration postgastrectomy but also on early Rikkunshito administration in the postoperative phase as being the key time for producing beneficial clinical effects.

In addition to promoting gastrointestinal motility, the mitigating effects of Rikkunshito on weight loss and overall nutritional status when administered early in the postoperative phase show the broad and multifaceted benefits of this herbal medicine. A holistic approach to patient management involves not only surgical techniques but also perioperative interventions aimed at preserving nutritional status and enhancing postoperative recovery. This concept is in accordance with the recommendations outlined in guidelines from authoritative bodies,^[32,33]^ underscoring the importance of integrating nutritional support into the routine perioperative care of gastric cancer patients.

Previous studies have also investigated the roles of nutritional supplements, dietary interventions, and pharmaceutical agents in mitigating postoperative complications and optimizing nutritional outcomes. Kobayashi et al^[34]^ and Sakuraya et al^[35]^ have demonstrated the effects of nutritional interventions on the postoperative outcomes of gastric cancer patients. Our present results complement their findings, offering insights into the potential roles of Rikkunshito in preserving nutritional status and preventing weight loss when administered at a fairly early stage after gastrectomy.

Our study prompts a deeper investigation into the specific mechanisms underlying the beneficial nutritional outcomes of Rikkunshito. Emerging research has investigated the molecular and physiological aspects of herbal interventions, revealing increased plasma ghrelin levels, inhibition of ghrelin metabolism, enhancement or upregulation of receptor sensitivity, and inhibition of excessive glucagon-like peptide-1 (GLP-1) elevation, leading to appetite stimulation and potential pathways involving nutrient absorption and inflammatory modulation.^[36–38]^ These studies contribute to a deeper understanding of the mechanisms of action of herbal medicines in the context of postoperative care. Our present findings add a valuable dimension to the body of literature exploring various perioperative interventions for gastric cancer patients.

## 5. Limitations

This present study has some limitations. This study used a single institution retrospective design, which may have led to bias in the results. The sample size of each group was small as PSM was performed to balance the baseline characteristics of both groups. Furthermore, since our study was retrospective and included all eligible patients during the study period, a priori power analysis was not feasible. However, as information for future research, we performed a post hoc power analysis based on our findings. Specifically, we observed the mean difference in postoperative body weight loss at 1 month between the Rikkunshito and control groups of approximately 3.0%, with an estimated standard deviation of 4.0%. Assuming a 2-sided α of 0.05 and a power of 0.80, a future prospective study would require approximately 29 patients per group (a total of 58 patients) to detect this difference. In this study, we aimed to identify the optimal timing of Rikkunshito administration postgastrectomy by measuring postoperative body weight loss and nutritional status. We were not able to correlate the effects of Rikkunshito on other factors, such as the plasma ghrelin and GLP-1 levels. Moreover, Rikkunshito consists of multiple active ingredients. However, we were not able to analyze which of these ingredients contributed most significantly to the observed effects. Future prospective investigations with larger sample sizes should be conducted to enhance the robustness of the findings and consolidate evidence regarding the efficacy of the early administration of Rikkunshito postgastrectomy as well as pharmacological investigations to elucidate the detailed underlying mechanisms of our findings. In terms of research and articles on the use of Kampo medicine (particularly Rikkunshito) in the perioperative period of gastrectomy, these remain scarce. Thus, a number of references cited in the present study are dated. It is hoped that the present study will provide new information on the use of Rikkunshito and contribute to future research on the importance of Rikkunshito and Kampo medicine in the perioperative phase of gastrectomy.

## 6. Conclusion

Early administration of Rikkunshito during the perioperative period may help alleviate postoperative weight loss and support nutritional recovery in patients undergoing minimally invasive distal gastrectomy for gastric cancer. These findings suggest a potential benefit of initiating Rikkunshito soon after surgery. Future studies including larger cohorts and evaluation of mechanisms such as plasma ghrelin dynamics are warranted to further validate and elucidate these effects.

## Author contributions

**Conceptualization:** Kenichi Iwasaki, Edward Barroga, Hiroyuki Koga, Yuichi Nagakawa.

**Data curation:** Kenichi Iwasaki, Edward Barroga, Yuki Takano, Toru Sakurai, Erika Yamada, Masaya Enomoto, Yota Shimoda, Junichi Mazaki.

**Investigation:** Kenichi Iwasaki, Edward Barroga, Yuki Takano, Toru Sakurai, Erika Yamada, Masaya Enomoto, Yota Shimoda, Junichi Mazaki.

**Methodology:** Kenichi Iwasaki, Yuki Takano, Toru Sakurai, Erika Yamada, Masaya Enomoto, Yota Shimoda, Junichi Mazaki.

**Supervision:** Kenichi Iwasaki, Hiroyuki Koga, Akishige Kanazawa, Yuichi Nagakawa.

**Validation:** Kenichi Iwasaki, Edward Barroga, Yuki Takano, Toru Sakurai, Erika Yamada, Masaya Enomoto, Yota Shimoda, Junichi Mazaki, Hiroyuki Koga, Akishige Kanazawa, Yuichi Nagakawa.

**Writing – original draft:** Kenichi Iwasaki, Edward Barroga.

**Writing – review & editing:** Kenichi Iwasaki, Edward Barroga.
